# Population Pharmacokinetic Analyses for Rezafungin (CD101) Efficacy Using Phase 1 Data

**DOI:** 10.1128/AAC.02603-17

**Published:** 2018-05-25

**Authors:** Elizabeth A. Lakota, Voon Ong, Shawn Flanagan, Christopher M. Rubino

**Affiliations:** a Institute for Clinical Pharmacodynamics, Inc., Schenectady, New York, USA; b Cidara Therapeutics, San Diego, California, USA

**Keywords:** echinocandin, pharmacokinetics, population pharmacokinetics

## Abstract

Rezafungin (CD101) is a novel echinocandin antifungal agent currently in clinical development for the treatment of candidemia and invasive candidiasis. Rezafungin has potent *in vitro* activity against Candida albicans and Candida glabrata, including azole- and echinocandin-resistant isolates. The objective of this analysis was to develop a population pharmacokinetic (PK) model to characterize the disposition of rezafungin in plasma following intravenous (i.v.) administration. Data from two phase 1 studies, a single-ascending-dose study and a multiple-ascending-dose study, were available. Candidate population PK models were fit to the pooled data using the Monte Carlo parametric expectation maximization algorithm in S-ADAPT. The data were best described using a linear four-compartment model with zero-order drug input via i.v. infusion and first-order elimination. In order to account for the relationships between the structural PK parameters and subject body weight, all parameters in the model were scaled to subject body weight using standard allometric coefficients (a power of 0.75 for the clearance terms and 1.0 for the volume terms). The final model fit the observed data with very little bias and excellent precision. The prediction-corrected visual predictive check demonstrated that the final model could accurately simulate both the central tendency and the variability of observed rezafungin plasma concentrations. Given this, the final rezafungin population PK model is expected to provide reliable simulated concentration-time profiles and can provide dose selection decision support for future clinical studies.

## INTRODUCTION

Historically, Candida albicans has been the predominant pathogen in candidemia and invasive candidiasis infections (1). Despite this, Candida glabrata is now emerging as a greater concern for clinicians due to a recent increase in prevalence among candidemia and invasive candidiasis infections and the pathogen's natural predisposition for expressing azole and echinocandin resistance mutations (2, 3). Accordingly, the incidence of infections due to C. glabrata not only has increased more than 4-fold over the past 20 years (4) but also has been accompanied by an increased prevalence of bloodstream infections due to resistant C. glabrata isolates (5, 6). Rezafungin (CD101) is a novel echinocandin antifungal agent currently in development for the treatment and prevention of candidemia and invasive candidiasis. Rezafungin has potent *in vitro* activity against C. albicans and C. glabrata, including azole- and echinocandin-resistant isolates. In addition, rezafungin has shown *in vivo* activity against a range of C. albicans and C. glabrata isolates, including, importantly, an echinocandin-resistant C. glabrata isolate (7).

One of the unique features of rezafungin is its long half-life. Analyses of phase 1 single- and multiple-ascending-dose studies showed that the rezafungin half-life in humans is approximately 133 h (5.5 days) (8). This value far exceeds those reported for other echinocandins, which have terminal half-lives ranging from 9 to 52 h (9–11). The rezafungin maximum concentration, area under the concentration-time curve (AUC), and half-life were shown to be dose proportional up to 400 mg, the highest dose evaluated in the studies (8). Given rezafungin's long half-life, it is being evaluated in clinical studies using dosing regimens with a once-weekly dosing frequency. The aforementioned analyses of phase 1 data found minor accumulation (30 to 55%) of rezafungin following weekly dosing. Moreover, no safety signals were noted in these studies. Of the 42 patients evaluated, only 4 experienced moderate treatment-related adverse events. No serious or severe adverse events were observed in either study.

As the prior analyses performed were conducted using noncompartmental techniques, a quantitative model describing the time course and variability of rezafungin concentrations in humans was desired to facilitate Monte Carlo simulations to aid in dosing regimen selection for future clinical studies. Our objective was to develop a population pharmacokinetic (PK) model to characterize the disposition of rezafungin in plasma following intravenous (i.v.) administration.

## RESULTS

### Pharmacokinetic analysis data set.

The final data set consisted of 840 rezafungin samples collected from 42 healthy subjects across two studies. All concentrations were above the lower limit of quantification. No outliers were identified during the population PK analysis. Out of the 485 samples collected from subjects in the multiple-ascending-dose study, 257 (53.0%) were collected after administration of multiple rezafungin infusions. Table 1 presents demographic and select laboratory measures for the subjects in the analysis data set. The majority of the subjects were Caucasian (90.4%) and male (52.3%). Subjects ranged in age from 22 to 54 years. The mean weight observed was 76.2 kg (minimum, 57.1 kg; maximum, 102 kg). The typical subject was overweight, as indicated by a mean body mass index (BMI) of 27.7 kg/m^2^. All subjects had normal renal function (minimum creatinine clearance [CL_CR_], 80 ml/min/1.73 m^2^).

**TABLE 1:** Summary statistics for demographics and clinical laboratory measures among subjects administered rezafungin

Characteristic	No. (%) of subjects	Mean (SD) value	Median value	Minimum value	Maximum value
Age (yr)	42	41.7 (8.95)	42.5	22	54
Wt (kg)	42	76.2 (11.1)	75.0	57.1	102
Ht (cm)	42	166 (8.74)	165	148	187
BSA (m^2^)	42	1.84 (0.17)	1.81	1.51	2.22
BMI (kg/m^2^)	42	27.7 (2.76)	27.8	22.4	31.9
CL_CR_ (ml/min/1.73 m^2^)	42	112 (17.9)	113	79.6	153
Albumin (mg/dl)	42	4.46 (0.24)	4.50	4.00	4.80
Race					
Caucasian	38 (90.4)				
Black	3 (7.14)				
Other	1 (2.38)				
Sex					
Male	22 (52.3)				
Female	20 (47.6)				

### Population pharmacokinetic model development.

**FIG 1:**
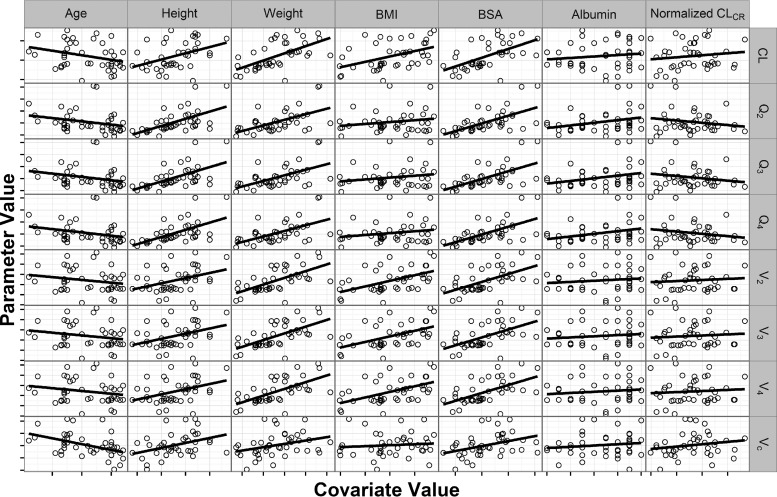
Scatterplots of individual parameter estimates versus covariate values using the structural population PK model. Abbreviations: BMI, body mass index; BSA, body surface area; CL, total clearance; *V_c_*, volume of distribution of the central compartment; *Q*_2_, *Q*_3_, and *Q*_4_, distributional clearances; *V*_2_, *V*_3_, and *V*_4_, volume of distribution of the peripheral compartments; CL_CR_, creatinine clearance.

Linear two-, three-, and four-compartment models were evaluated during structural model development. Goodness-of-fit plots for the three- and four-compartment models are displayed in Fig. S1 and S2 in the supplemental material, respectively. The addition of a fourth compartment resulted in less biased residual versus time since last dose plots and a 55-unit decrease in the objective function. Given these findings, a linear four-compartment model with zero-order drug input via the i.v. infusion and first-order elimination was selected as the base structural model. The results of the graphical exploration of the continuous covariates versus individual model parameter estimates are displayed in Fig. 1. The strongest relationship found (correlation coefficient = 0.622) was between weight and the volume of the first peripheral compartment (*V*_2_). Weight was moderately correlated with all eight model parameters (correlation coefficients ≥ 0.230). Weight was selected for testing in the model, given its physiologic plausibility, correlation with all model parameters, and widely accepted standard allometric scaling coefficients for both volume and clearance terms. The weight relationship was implemented using a power function centered on 75 kg. The power coefficients were fixed to standard estimates of 0.75 for the clearance terms and 1.0 for the volume terms (12). Addition of weight to the model decreased the objective function by 8.41 units, a statistically significant decline considering that no additional model parameters were estimated. Addition of weight also explained 5% of the interindividual variability on both total clearance and the volume of distribution of the central compartment. Given these model improvements, the weight relationships were included in the final model.

### Final population pharmacokinetic model.

**FIG 2:**
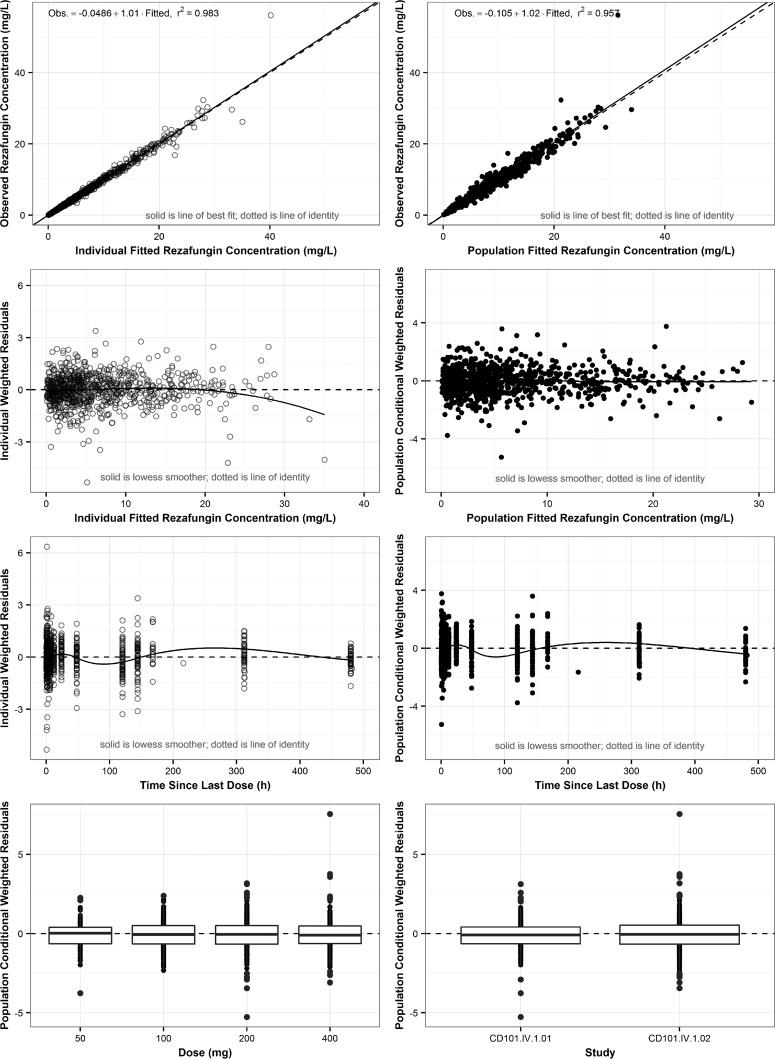
Rezafungin plasma goodness-of-fit plots using the final population PK model.

Final population PK parameter estimates and their associated precision for rezafungin are provided in Table 2. The individual and population predicted rezafungin concentrations were unbiased and agreed well with the observed data (*r*^2^ = 0.98 and 0.96, respectively; Fig. 2). There was a lack of bias when residuals were plotted by predicted concentration, time, dose, and study, as displayed in Fig. 2. For a typical subject weighing 75 kg, rezafungin clearance was estimated to be 0.187 liter/h, while typical 50- and 100-kg subjects were estimated to have rezafungin clearances of 0.138 and 0.232 liter/h, respectively.

**TABLE 2:** Rezafungin population PK model parameter estimates and standard errors^*a*^

Parameter	Population mean		Magnitude of IIV (CV %)	
	Final estimate	SEM (%)	Final estimate	SEM (%)
CL (liter/h)	0.188		13.0	14.6
*V_c_* (liters)	8.94		28.0	15.6
*Q*_2_ (liters/h)	24.4		45.2	17.1
*V*_2_ (liters)	12.6		8.81	17.0
*Q*_3_ (liter/h)	0.912		84.3	17.0
*V*_3_ (liters)	8.75		19.6	15.9
*Q*_4_ (liter/h)	0.0739		44.7	16.3
*V*_4_ (liters)	27.9		81.3	18.0
CL coefficient (liter/h/75 kg)	0.187	1.46		
*V_c_* coefficient (liters/75 kg)	8.88	3.22		
*Q*_2_ coefficient (liters/h/75 kg)	24.3	5.42		
*V*_2_ coefficient (liters/75 kg)	12.5	1.04		
*Q*_3_ coefficient (liter/h/75 kg)	0.908	10.0		
*V*_3_ coefficient (liters/75 kg)	8.70	2.35		
*Q*_4_ coefficient (liter/h/75 kg)	0.0736	5.29		
*V*_4_ coefficient (liters/75 kg)	27.7	9.70		
SD_in_	0.01	Fixed		
SD_sl_	0.0624	4.72		

a The minimum value of the objective function was −488.108. Abbreviations: CL, total clearance; *V_c_*, volume of distribution of the central compartment; *Q*_2_, *Q*_3_, and *Q*_4_, distributional clearances; *V*_2_, *V*_3_, and *V*_4_, volume of distribution of the peripheral compartments; SD_in_, intercept (additive) term for residual variability model for plasma concentrations; SD_sl_, slope (proportional) term for residual variability model; IIV, interindividual variability; CV, coefficient of variation; SEM, standard error of the mean.

**FIG 3:**
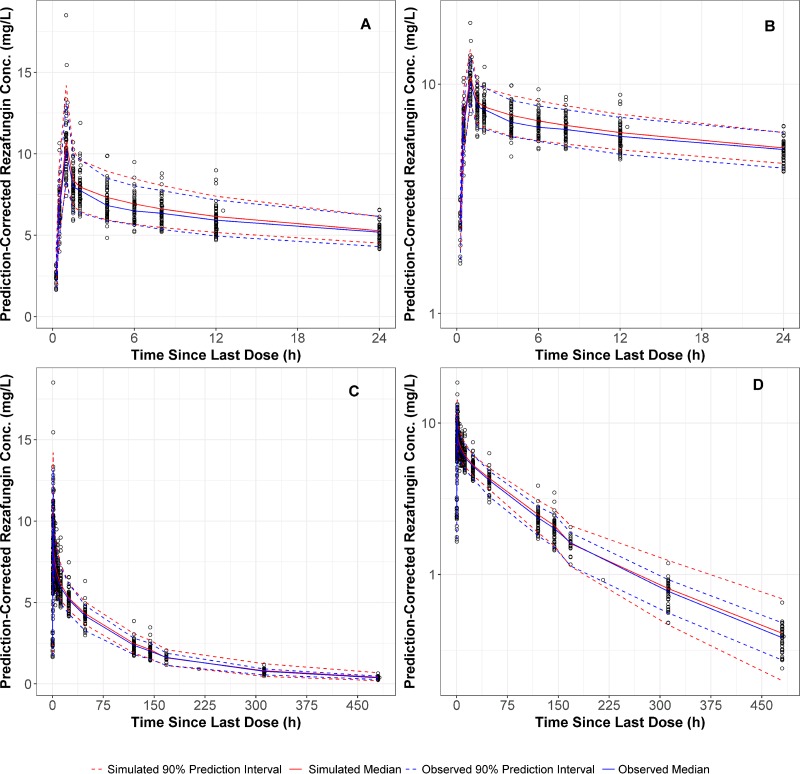
Prediction-corrected comparison of observed and simulated rezafungin plasma concentrations on linear (A and C) and semilog (B and D) scales up to 24 h (A and B) and 480 h (C and D) postdose.

The prediction-corrected visual predictive check (PC-VPC) plot for the final rezafungin population PK model is provided in Fig. 3. The majority of the observed concentrations were contained within the 90% prediction interval. Additionally, the observed median and 90% prediction interval were very similar to the simulated median and 90% prediction interval, suggesting that the final population PK model provided an accurate and unbiased fit of the rezafungin PK data.

## DISCUSSION

A population PK model was successfully developed to describe rezafungin plasma concentration-time data following i.v. administration of single and multiple rezafungin doses in healthy volunteers. The final model was a linear four-compartment model with all parameters scaled to subject body weight using standard allometric scaling coefficients. The final model is structurally similar to population PK models for anidulafungin, caspofungin, and micafungin (13–15). All three of these models are linear, multicompartment models with weight as a covariate on clearance. Weight is also a covariate on select volume terms in the anidulafungin and caspofungin models. Linear, multiphasic PK impacted by weight appears to be a consistent characteristic of the echinocandin class, including rezafungin.

During the covariate evaluation step, BMI, body surface area (BSA), and height were also correlated to several model parameters, signaling the consistent impact of body size on rezafungin PK. Although the correlations were not as strong as those observed with body weight, another body size measure may also adequately describe variability in various rezafungin PK parameters. The data set utilized for this analysis had a limited number of subjects (*n* = 42). In addition, the subjects were fairly homogeneous, as evidenced by the low coefficient of variation (CV) observed for body weight (15%) relative to those reported previously (21 to 32%) among infected patients (15–18). The small homogeneous data set is a limitation of the current analysis. Given this, the model will need to be refined and a formal covariate analysis will be needed once more data are available. The updated covariate analysis will be especially critical once data from special populations (e.g., obese patients) and phase 2 and 3 studies, which enroll large numbers of patients with greater covariate variability, are available.

The model-predicted mean rezafungin clearance for study subjects was 0.188 liter/h, consistent with the clearance values reported from the noncompartmental analysis, which ranged from 0.126 liter/h to 0.279 liter/h (8). This is considerably lower than the model-estimated mean clearances for anidulafungin, caspofungin, and micafungin, which range from 0.411 liter/h to 1.22 liter/h (13–15). A lower clearance suggests that higher AUC values can be achieved using the same dose, which is critical, given that the ratio of AUC to MIC drives efficacy for this agent (19). Moreover, the model-estimated interindividual variability on rezafungin clearance (13%) was lower than that of other echinocandins, which ranges from 28% to 36% (13–15). However, the population PK models for anidulafungin, caspofungin, and micafungin were developed using data from infected patients enrolled in phase 2 and 3 studies. PK in infected patients is often more variable than that in healthy volunteers (16, 20), further supporting the need for an updated, formal covariate analysis once phase 2 data are available.

As shown in Fig. 3, simulations conducted using the final model could accurately estimate both the central tendency and the variability of the observed rezafungin plasma concentrations. This indicates that both the model population mean and interindividual variability parameter estimates are reliable. Therefore, the final population PK model is expected to provide dependable simulations of rezafungin concentration-time profiles following administration of rezafungin at doses of up to 400 mg in subjects with similar demographics to the current data set. The final model can be utilized to conduct Monte Carlo simulations of various dosing regimens to provide dose selection decision support for future clinical studies.

## MATERIALS AND METHODS

### Studies.

Data were pooled from two phase 1 studies (ClinicalTrials.gov registration no. NCT02516904 and NCT02551549) previously described by Sandison et al. (8). The protocols and informed consent were reviewed and approved by an appropriate institutional review board before subject enrollment. The studies were designed and monitored in compliance with the ethical principles of good clinical practice and in accordance with the Declaration of Helsinki.

The first study (ClinicalTrials.gov registration no. NCT2516904) evaluated single i.v. doses of rezafungin. A total of 32 subjects were enrolled and randomized evenly to one of four cohorts. Within each cohort, six subjects were administered rezafungin and two subjects were administered placebo as a single 60-min i.v. infusion. A single rezafungin dose of 50, 100, 200, and 400 mg was administered to cohorts 1, 2, 3, and 4, respectively. In all cohorts, blood samples for PK evaluations were collected predose and at 0.25, 0.5, 1, 1.5, 2, 4, 6, 8, 12, 24, 48, and 120 h after the start of infusion and on days 7, 14 (±1 day), and 21 (±1 day).

The second study (ClinicalTrials.gov registration no. NCT02551549) evaluated multiple i.v. doses of rezafungin. A total of 24 subjects were enrolled and randomized evenly to one of three cohorts. Within cohorts 1 and 2, six subjects were administered once-weekly i.v. doses of rezafungin at 100 and 200 mg, respectively, and two subjects were administered once-weekly i.v. doses of placebo, infused over 60 min on days 1 and 8. Within cohort 3, six subjects were administered once-weekly i.v. rezafungin doses of 400 mg and two subjects were administered once-weekly i.v. doses of placebo, infused over 60 min on days 1, 8, and 15. Blood samples were collected predose and at 0.5, 1, 1.5, 2, 4, 6, 8, 12, 24, 48, and 120 h after the start of each infusion and on days 7, 14 (±1 day), 21 (±1 day), and 35 (±1 day, cohort 3 only).

### Analytical methods.

All blood samples were collected using tubes with K_2_-EDTA. Plasma was collected by centrifugation and stored at −70°C until analysis. Samples were analyzed using a 100-μl aliquot volume and a protein-precipitation extraction procedure followed by liquid chromatography/tandem mass spectrometry analysis. The rezafungin calibration range was 10.0 ng/ml to 10,000 ng/ml, using d9-CD101 as an internal standard. The range of the assay was extended 10-fold by dilution. An AB-Sciex API 4000 system was operated in the selected reaction-monitoring mode under optimized conditions for detection of rezafungin and d9-CD101 positive ions formed by electrospray ionization. Excellent interassay accuracy and precision, as measured by the use of quality control (QC) samples, were obtained during validation. Across the range of QC samples tested (lower limit of quantitation through the high-QC range), the interassay accuracy (percent bias from nominal) ranged from 0.3% to 4.3%, while the interassay precision (percent CV) ranged from 2.7% to 5%. Dilution QC samples performed well, with −1.5% bias and an interassay precision of 3.4%. Rezafungin was found to be stable through four freeze-thaw cycles, as well as at room temperature for at least 24 h. Under frozen storage conditions, rezafungin was found to be stable for at least 164 days either at −20°C or at −70°C.

### General data handling.

The actual dates and times of dose administration and PK sample collection were used in the construction of the population PK data set. Subjects who received placebo were not included in the data set. An outlier was defined as an aberrant observation that substantially deviated from the rest of the observations within an individual. Any suspected outliers were to be tested and, if justified, excluded from this analysis, given the potential for these observations to negatively impact the convergence and/or parameter estimates (21).

### Population pharmacokinetic modeling.

Candidate population PK models were fit to the pooled data using Monte Carlo parametric expectation maximization as implemented in the open-source software program S-ADAPT (version 1.5.6) (22–24). The model selection criteria used to discriminate between candidate PK models included (i) evaluation of individual and population mean parameter estimates and their precision (standard error of the mean [SEM]), (ii) graphical examination of standard goodness-of-fit plots and plots of the observed versus individual predicted concentrations, (iii) reductions in both residual variability and interindividual variability, and (iv) comparison of the objective function for nested models or the Akaike information criterion (25) for either nested or nonnested models.

Interindividual variability was estimated for each structural population PK model parameter, where possible, by using an exponential-error model. Residual variability was initially described by using an additive plus proportional CV error model. Other models for residual variability were explored as necessary. After development of the structural model, relationships between patient-specific covariates and individual *post hoc* parameter estimates were evaluated via graphical exploration. The covariates evaluated included age, albumin, creatinine clearance (CL_CR_), and various measures of body size (weight, height, BMI, and BSA). Covariates with strong relationships were tested in the population PK model.

The final population PK model was qualified by performing a PC-VPC, which examines the agreement between the 5th, 50th, and 95th percentiles of the observed and the individual simulated concentrations across time intervals. This procedure uses the original analysis data set as a template to simulate PK data for the same number of subjects in 500 new data sets, with each data set featuring the same study design and data collection scheme. The PC-VPC normalizes both the observed and the simulated concentration-time data by the median population predictions during each time interval to adjust for the differences due to independent variables in the final population PK model, thus avoiding the need to stratify by single- versus multiple-dose data, dose group, or other significant covariate effects included in the model.

## Supplementary Material

Supplemental material
