# Supplementary material for: Population Pharmacokinetic Analyses for Rezafungin (CD101) Efficacy Using Phase 1 Data
Source: Antimicrob Agents Chemother. 2018 May 25;62(6):e02603-17. doi: 10.1128/AAC.02603-17 (PMC5971566; doi:10.1128/AAC.02603-17)
Supplement: Supplemental material [file supp_62_6_e02603-17__index.html]

Supplemental material 

# Population Pharmacokinetic Analyses for Rezafungin (CD101) Efficacy Using Phase 1 Data

## Supplemental material

- Supplemental file 1 -

  Supplemental Figures S1 and S2

  PDF, 530K
