# Supplementary material for: Population Pharmacokinetic Analyses for Rezafungin (CD101) Efficacy Using Phase 1 Data
Source: Antimicrob Agents Chemother. 2018 May 25;62(6):e02603-17. doi: 10.1128/AAC.02603-17 (PMC5971566; doi:10.1128/AAC.02603-17)

**Supplemental Figure 1**. Rezafungin plasma goodness-of-fit plots for the linear three-compartment model

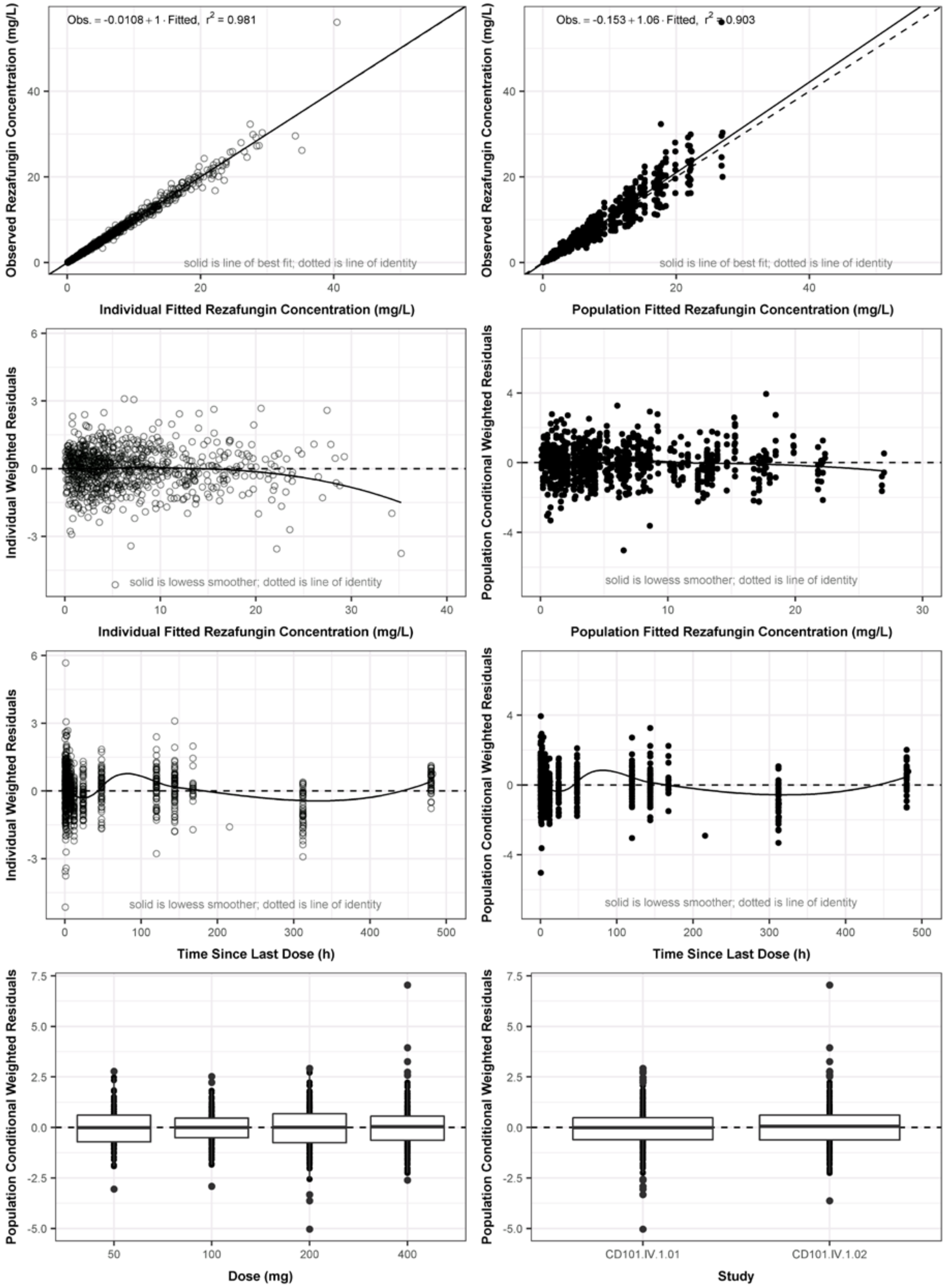

**Supplemental Figure 2**. Rezafungin plasma goodness-of-fit plots for the linear four-compartment model

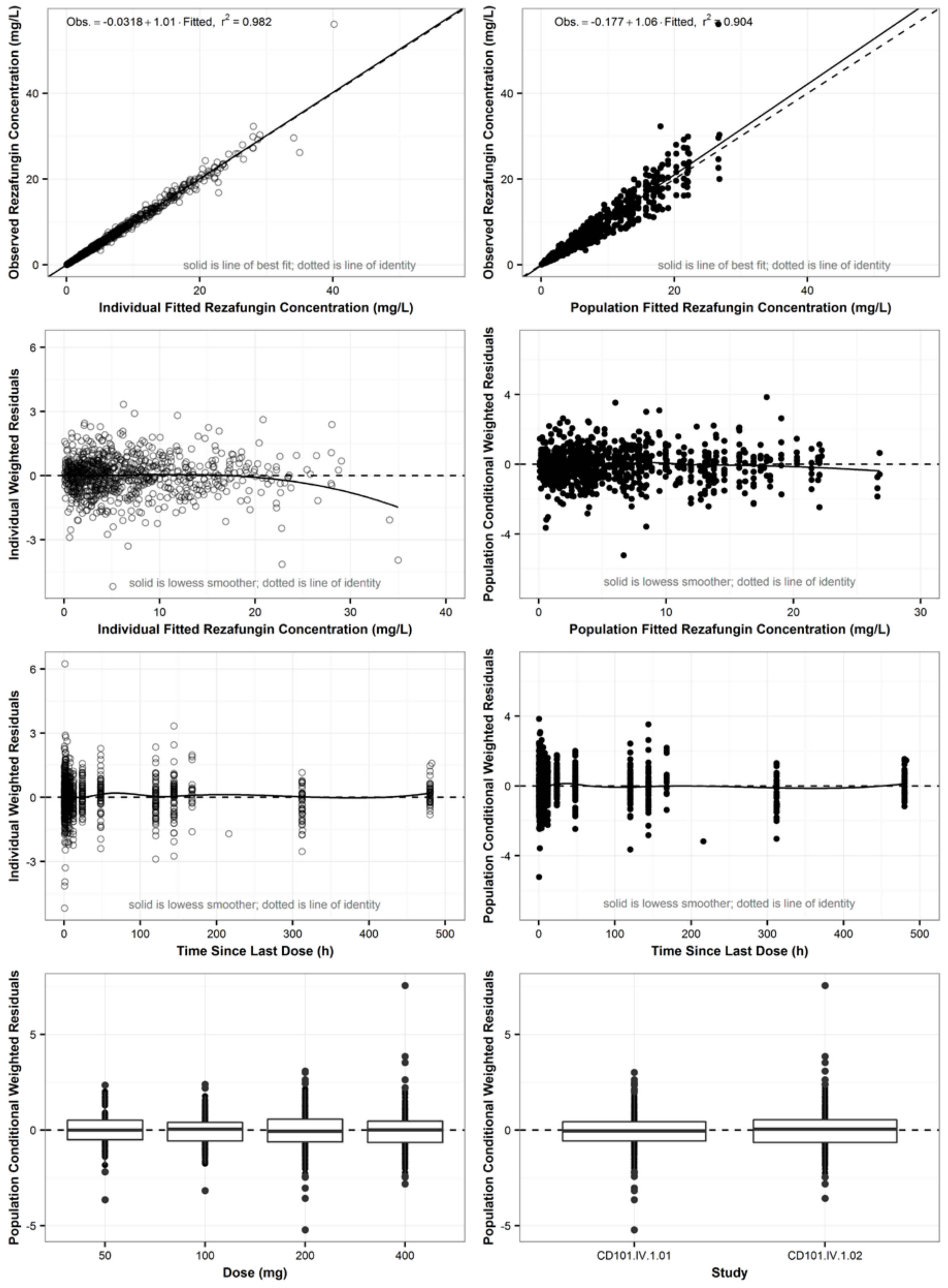

Supplement: Supplemental material [file AAC.02603-17_zac006187177s1.pdf]
